# Characterization of a not so new potexvirus from babaco (*Vasconcellea x heilbornii*)

**DOI:** 10.1371/journal.pone.0189519

**Published:** 2017-12-15

**Authors:** Robert A. Alvarez-Quinto, Juan F. Cornejo-Franco, Diego F. Quito-Avila

**Affiliations:** 1 Centro de Investigaciones Biotecnológicas del Ecuador, CIBE, Escuela Superior Politécnica del Litoral, ESPOL, Guayaquil, Ecuador; 2 Facultad de Ciencias de la Vida, Escuela Superior Politécnica del Litoral, ESPOL, Guayaquil, Ecuador; Oklahoma State University, UNITED STATES

## Abstract

A new member of the genus *Potexvirus* was fully sequenced and characterized. The virus was isolated from babaco (*Vasconcellea x heilbornii*), a natural hybrid native to Ecuador. The virus contains a 6,692 nt long genome organized in five open reading frames in an arrangement typical of other potexviruses. Sequence comparisons revealed close relatedness with *Papaya mosaic virus* (PapMV), *Alternathera mosaic virus* (AltMV) and *Senna mosaic virus* (SenMV), exhibiting nucleotide identities up to 67% for the polymerase (Pol) and 68% for the coat protein (*CP*), with deduced amino acid identities of 70% and 72% for the Pol and CP, respectively. The presence of an AlkB domain, in the polymerase region, was observed. Terminal nucleotide sequences were conserved across potexviruses with characteristic motifs and predicted secondary structures at the 3’ UTR. Although serologically undistinguishable from PapMV and AltMV, the new virus showed differences in host range and symptom induction. The name babaco mosaic virus is proposed for this newly characterized *Potexvirus*. The complete genome sequence of the new virus has been deposited in NCBI GenBank under accession number MF978248.

## Introduction

Babaco (*Vasconcellea x heilbornii*) is a natural interspecific hybrid of *V*. *stipulata* and *V*. *pubescens* in the family *Caricacea*. The plant, formerly known as *Carica pentagona* due to its peculiar pentagon-shaped fruits, is an herbaceous shrub that can grow up to 2.5 meters in height and has morphological characteristics similar to those of its close relative papaya (*Carica papaya*). Although native to subtropical mountains of southern Ecuador, babaco has been introduced and grown at small scale in Australia, New Zealand, United States, Italy and Spain, among others. In addition to its particularly exotic flavour, the babaco latex has been demonstrated to have lipase activity higher than that of papaya, showing its potential industry use [1].

In the last two decades, babaco demand for local fresh market has increased considerably and its production has become the main income source for hundreds of small growers in different provinces of Ecuador. However, the expansion in cultivated area, along with the vegetative propagation nature of this crop, have resulted in the emergence of several pathogen related diseases that hamper the sustainable and profitable production of this exotic fruit [2,3]

While several fungal diseases have been identified and characterized in babaco, studies on disease-causing viruses have been overlooked. In 1989, potexvirus-like particles were identified and purified from babaco plants showing yellow mosaic symptoms in Italy [4]. The virus was shown to be serologically related to *Papaya mosaic virus* (PapMV) [5]. To the best of our knowledge, no further work was done to characterize the virus.

In this study, a new potexvirus was characterized. The virus was isolated from babaco orchards in Ecuador and considering the introduction of Ecuadorean babaco to Italy in the early 80’s, we speculate that the virus is an isolate of the one reported and partially studied in Italy in 1989 [4].

## Materials and methods

### Plant material and virus isolate maintenance

Leaves from babaco plants showing leaf mottling and mosaic were collected, with owners consent, from small orchards in subtropical regions of Azuay and Santo Domingo provinces. Leaves were ground in phosphate buffer and mechanically inoculated onto papaya cv. ‘Sunrise’ plants. Inoculated plants were maintained under standard greenhouse conditions.

### Double-stranded RNA extraction, cloning and sequencing

Twenty-gram batches were used for double-stranded RNA (dsRNA) extraction using a cellulose-based protocol for detection of RNA viruses in plants [6]. The dsRNA was heat-denatured and used as template for shotgun sequencing by degenerate oligonucleotide-primed (DOP) RT-PCR as described [7]. Amplification products from DOP-RT-PCR were cloned using a TOPO-TA cloning kit (Life technologies, USA) and sequenced at Macrogen (Seoul, Korea). Sequence reads were assembled into contigs using Geneious^®^ 8.2.1 [8]. Contigs were subjected to blast analysis to determine the closest hit available in the NCBI database. Once the reference sequence (closest relative) was identified, primers were designed to fill genome gaps. The 5’ end was obtained by poly A tailing on cDNA using terminal transferase and specific primers at the end of the terminal regions. The first nucleotide was re-confirmed applying the same procedure, but using poly C tailing instead [9,10]. The 3’ end was obtained by taking advantage of the viral poly A-tailed RNA using anchored oligo dT and specific primers.

### Genome organization and phylogenetic analysis

Genome organization including identification of characteristic genomic features were done using the open reading frame (ORF) finder available at NCBI and motif search tool from Geneious^®^ 8.2.1. For phylogenetic inferences, complete genome sequences for all available potexviruses were obtained from the NCBI database. Sequence alignments were performed using MUSCLE and for phylogenetic inferences, the maximum likelihood method plugged in MEGA^®^ 7 was applied using the most suited substitution models determined also by MEGA 7.

### Detection

Virus detection was done by RT-PCR using total RNA as described by Halgren et al. [11]. RT was performed using random hexamers and RevertAid^®^ reverse transcriptase (Thermo Scientific, USA) following manufacturer’s instructions. PCR was carried out in a 10 μL mixture containing 1 μL 10× buffer, 0.2 uL of 10 mM dNTPs, 1.5 μL cDNA template, 6.8 uL of molecular biology grade water, 0.1 μL of Taq DNA polymerase (GenScript, USA) and 0.2 μL of each primer (40 μM) designed and selected based on specificity across closest relatives. PCR parameters were as follows: 94°C for 4 min, 40 cycles of 94°C for 45 s, 57°C for 30 s and 72°C for 45 s, and a final extension step of 10 min at 72°C.

In addition, a commercial ELISA kit (Agdia, USA) developed for the detection of PapMV and AltMV was used to test for serological compatibility.

### Host range

Mechanical inoculations were done by dusting carborundum (600 mesh) on test leaves followed by rubbing infected tissue homogenized in phosphate buffer (0.05 M pH: 7.4). Test plants (5 plants for each species) included the following species: papaya (*Carica papaya*), chamburo (*Vasconcellea pubescens)*, tamarillo (*Solanum betaceum*), *Nicotiana benthamiana*, *Chenopodium quinoa*, *Chenopodium amaranticolor*, *Amaranthus dubius*, *Senna occidentalis*, *Senna alata*, *Cucurbita ecuadoriensis*, *Luffa operculata* and *Luffa cylindrica*.

## Results

### Sequence assembly

A single band of approximately 7 kb was observed in dsRNA preparations obtained from symptomatic samples but not from symptomless ones.

Approximately 80 sequence reads were assembled into five contigs showing sequence homology to several members of the potexvirus genus, with *Alternathera mosaic virus*, AltMV, as the closest relative. Consequently, AltMV genome was used as reference to map the contigs. The complete sequence of the newly assembled genome consisted of 6,692 nt excluding the poly A tail, which is characteristic of potexviruses. The complete annotated sequence was deposited in NCBI Genbank under acc. number MF978248.

### Genome organization and phylogenetic analysis

**Fig 1:**
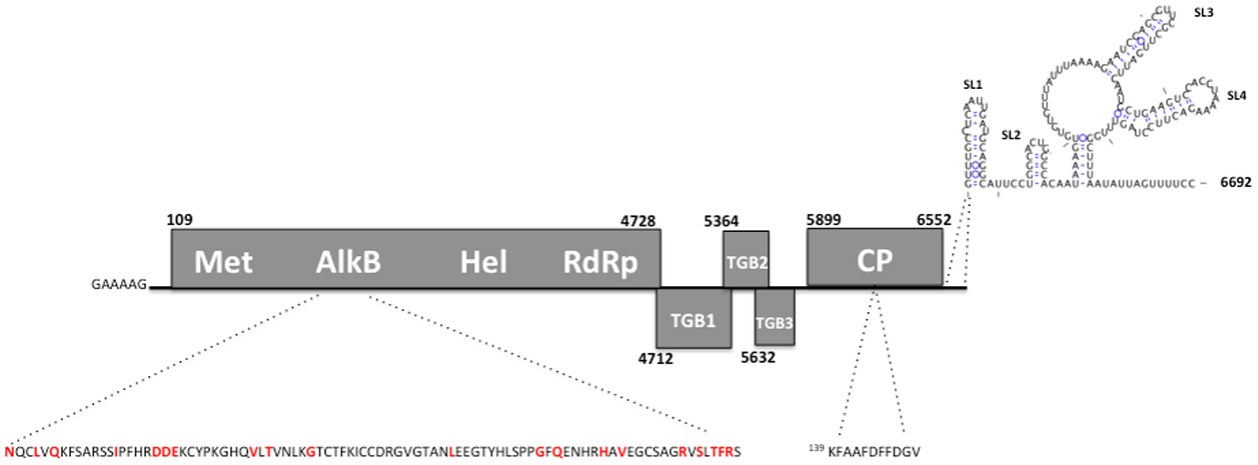
Genome organization of babaco mosaic virus. Predicted open reading frames (ORFs) with nucleotide coordinates are represented by rectangular boxes. Conserved motifs for methyltransferase (Met), AlkB, Helicase (Hel) and RNA-dependent-RNA polymerase (RdRp) are shown in ORF 1; where the core AlkB region is highlighted with assumed key functional residues shown in red. The triple gene block (TGB) proteins 1, 2 and 3 are denoted by TGB1, 2 and 3, respectively, in the overlapping ORFs 2, 3 and 4. The putative coat protein (CP) is shown in ORF 5 with the conserved potexvirus motif highlighted at amino acid (aa) position 139. Predicted secondary structures at the 3’ untranslated region (UTR) are shown as stem loops (SL).

The genome is organized in five ORFs similar to other potexviruses. ORF1 ^(nt 109–4,728)^ encodes the putative polymerase. ORF2 ^(nt 4,712–5,401)^, ORF3 ^(nt 5,364–5,708)^ and ORF4 ^(nt 5,632–5,838)^, arranged in an overlapping fashion, code for the movement-involved triple gene block (TGB) proteins 1, 2 and 3, respectively. Lastly, ORF5 ^(nt 5,899–6,552)^ encodes the putative coat protein (CP) (Fig 1).

Several genomic signatures have been observed across all members of the *Potexvirus* such as the overlapping TGB ORFs, highly conserved motifs, and the poly A tail, among others [12–14]. However, the presence of an AlkB domain—Fe (II)/2-OG-dependent dioxygenases involved in nucleic acid repair—in the replicase gene seems to distinguish two lineages in the evolutionary history of potexviruses. AlkB homologues have been reported in prokaryotes, eukaryotes and a few virus groups, being the *Flexiviridae* the one with more AlkB-containing members discovered thus far [15,16]. As shown in Fig 1, an AlkB domain was detected in the new babaco virus genome. The presence of highly conserved residues, which have been shown to be determinant in the RNA repair function [16, 17], support the notion that this domain may still be functional in this virus as opposed to be a remanent of an ancestral trait.

A highly conserved CP motif, involved in RNA-CP binding, has been reported as a signature genomic feature in potexviruses [18–24]. This motif was found in the new virus between aa positions 139–149 (KFAAFDFFDGV) (Fig 1) and was identical to the corresponding motifs in AltMV, SenMV and PapMV.

**Fig 2:**
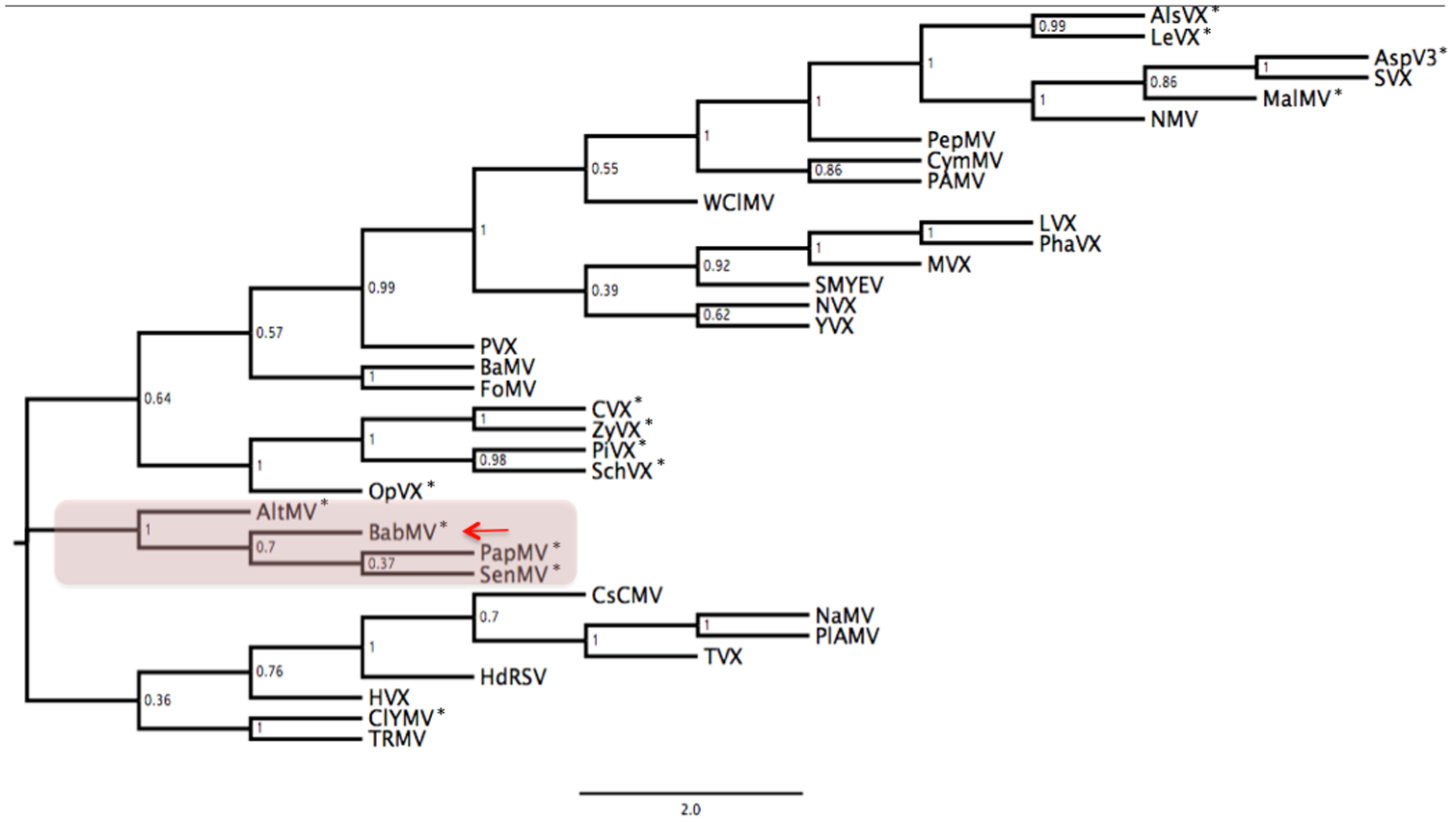
Polymerase based maximum-likelihood phylogenetic inference across members of the *potexvirus* genus. Clade containing the new babaco mosaic virus (BabMV) (indicated by the arrow) and its closest relatives: *Alternantera mosaic virus* (AltMV), *Papaya mosaic virus* (PapMV) and *Senna mosaic virus* (SenMV) is highlighted. Bootstrap values (1000 replicates) are shown in each node. Members with an AlkB domain are indicated by an asterisk. Complete names for each acronym, along with the corresponding NCBI acc. numbers are provided as S1 Table.

Phylogenetic inferences based on the complete polymerase across 36 potexvirus members showed that the new babaco virus clusters with AltMV, *Papaya mosaic virus* (PapMV) and *Senna mosaic virus* (SenMV) in a clade that is related closely to *Clover yellow mosaic virus* (ClYMV) and other AlkB containing members isolated from cactaceous hosts such as *Opuntia mosaic virus*, *Cactus virus X*, *Zygocatus virus X*, *Pitaya virus X* and *Schlumberga virus X*. Interestingly, four other AlkB containing members (*Alstroemeria virus X*, *Lettuce virus X*, *Malva mosaic virus* and *Asparagus virus 3*) were placed in a clade considerably distant from the other AlkB-containing species (Fig 2). When the AlkB region, detected in 14 out of 36 genomes, was removed from the alignment the tree topology was not altered.

Analysis of the CP sequence confirmed the evolutionary relationships across the potexvirus genus, where the new babaco virus formed a clade with PapMV, SenMV and AltMV, sharing a recent ancestor with the group of cactaceous viruses (S1 Fig).

Pairwise sequence comparisons between the genome of the new babaco virus and its closest relatives: AltMV, PapMV and SenMV, respectively, revealed nucleotide identities ranging from 65–67% for the polymerase and an average of 68% for the CP. At the amino acid level, an average of 70% identity was observed for the polymerase and 72% for the CP. The less conserved TGB proteins showed amino acid identities ranging from 55% to 63% for TGB1, 47% to 56% for TGB2 and 28% to 41% for the TGB3.

Based on ICTV guidelines for species demarcation in the genus *Potexvirus—*less than 70% nt identity and 80% aa level for either the polymerase or the CP—the new virus genome from babaco belongs to a distinct potexvirus species for which the name babaco mosaic virus (BabMV) is proposed and will be used hereafter for practical purposes.

Untranslated regions (UTR) of 108 nt and 140 nt were observed at the 5’ and 3’ ends, respectively. The hexanucleotide 5’-GAAAAG-3’ present at the 5’end of most potexviruses, was also observed in BabMV; whereas at the 3’ end, the pentanucleotide 5’-TTTCC-3’ showed conservation in BabMV and PapMV. Analysis of the 3’ UTR of BabMV revealed the presence of two conserved motifs 5’-^nt:6,593^AATAAA-3’ and 5’-^nt:6,652^ACCTAA-3’, which are putatively involved in signalling for poly (A) tailing and synthesis of negative-sense RNA in potexviruses [25–27]. In addition, at least three stem-loop structures were predicted on the 3’ UTR in BabMV similar to other potexviruses (Fig 1). Interestingly, the longer stem loop (SL) predicted in BabMV, possesses the hexanucletide motif 5’–ACCUAA–3’, which is also present in AltMV, SenMV and partially conserved in PapMV.

### Detection

Primers set: F: 5-GGATGCACTCATTACATCCAAGC-3 and R: 5’ CCACTCCAAGGCTTCCATGAGC-3’, which amplifies a 647 bp region of the polymerase, was selected based on specificity. The assay was tested on a set of 25 plants (n = 20 symptomless and n = 5 symptomatic) kindly provided by a commercial nursery in Azuay province. BabMV was detected in all symptomatic plants; whereas only one plant from the symptomless group, tested positive for the virus, but developed symptoms after three weeks. Similar results were obtained from ELISA testing, conducted in parallel. The presence of PapMV was not detected by RT-PCR in any of the tested plants, validating the cross-reactivity of PapMV-based antibodies with BabMV.

### Host range

**Fig 3:**
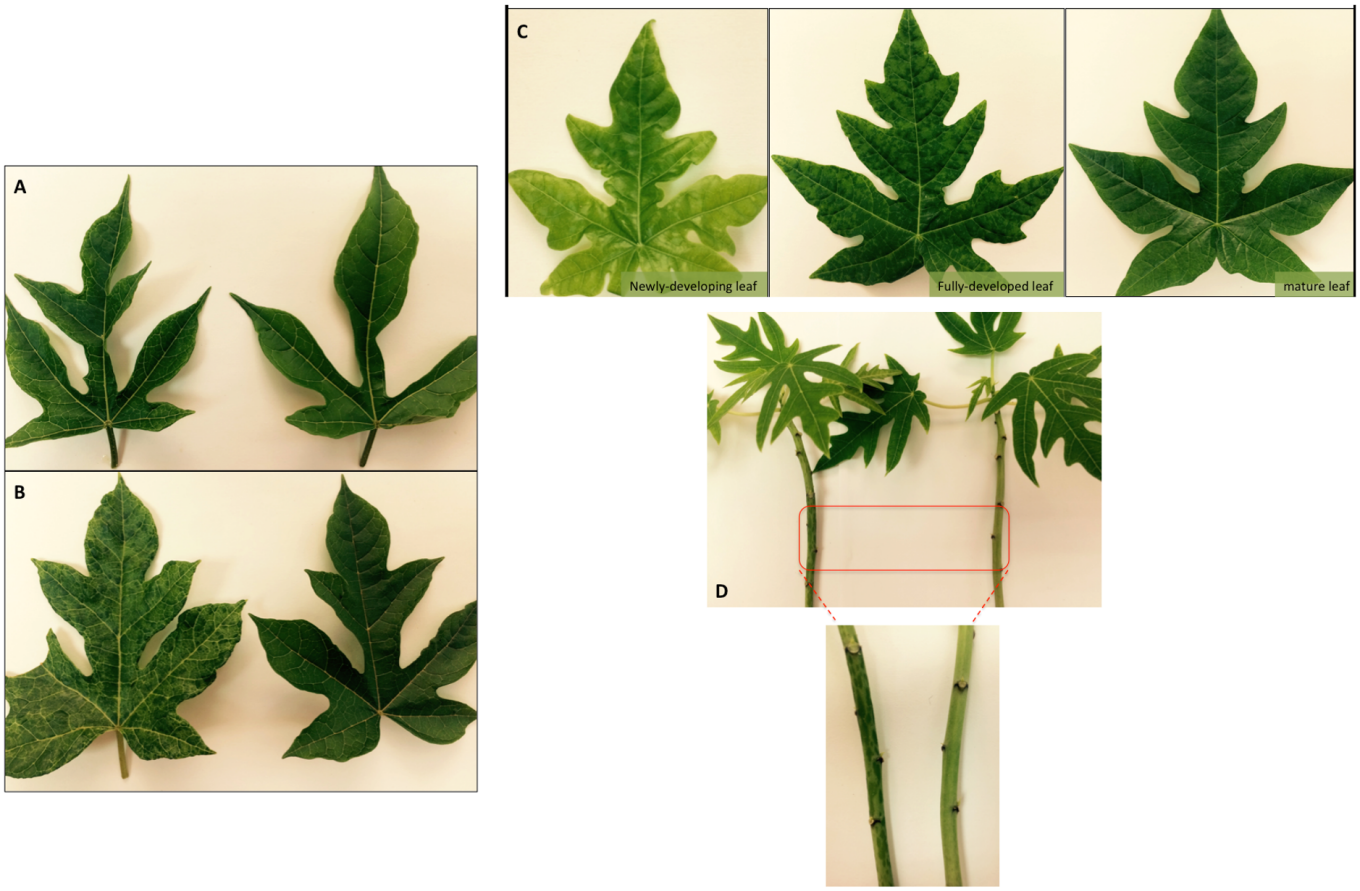
Symptoms induced by babaco mosaic virus. Leaves from non-inoculated plants are shown on the right and leaves from inoculated (symptomatic) plants are shown on the left. A) mild leaf mosaic observed in babaco, B) severe mosaic induced in chamburo, C) papaya leaves (from the same infected plant, cv. ‘Sunrise’) showing mosaic progression according to leaf maturity and D) water-soaked spots on papaya stems, cv. ‘Maradol’.

Two commercial papaya cultivars, ‘Maradol’ and ‘Sunrise’, were used for mechanical inoculations and virus maintenance. Symptoms were prominent in ‘Sunrise’, where a severe mosaic was visible on young leaves at 15 days post inoculation (dpi). Interestingly, as leaf growth progressed, symptoms vanished and plants remained symptomless until new fully developed leaves exhibited the mosaic (Fig 3). In ‘Maradol’, leaf mosaic was less pronounced but symptoms were maintained overtime; in addition, water-soaked spots were observed on the stems of this cultivar (Fig 3). In both cultivars, however, symptoms did not show a negative impact on plant growth and vigour during the course of this study (greenhouse conditions).

BabMV was back inoculated to symptomless virus-free babaco plants, where mild mosaic symptoms, similar to the ones originally observed, were visible at 25 dpi. Chamburo (*Vasconcellea pubescens)*, one of babaco’s parental lines, was also inoculated and showed severe leaf mosaic (Fig 3). In addition, the virus infected systemically, without inducing visible symptoms, the following hosts: *Chenopodium quinoa*, *Chenopodium amaranticolor* and S*enna occidentalis*.

BabMV was not detected on tamarillo (*Solanum betaceum*), *Nicotiana benthamiana*, *Amaranthus dubius*, *Cucurbita ecuadoriensis*, *Luffa operculata*, *Luffa cylindrical* and *Senna alata*.

## Discussion

The *Potexvirus—*a well-established genus in the *Alphaflexiviridae—*contains 35 officially recognized members found naturally in a variety of plant hosts [13,28]. Here, we report the genetic and biological characterization of a new member of the potexvirus isolated from symptomatic babaco. The virus, a ~ 500nm long filamentous particle (S2 Fig) for which the name babaco mosaic virus (BabMV) is proposed, exhibits close genetic relationships with *Papaya mosaic virus*, *Althernantera mosaic virus* and the recently characterized *Senna mosaic virus* [5,29,30]. Sequence comparisons between BabMV and its closest relatives, showed identity values below the threshold for species demarcation, according to ICTV guidelines for demarcation of potexviruses [12,31]. Phylogenetic inferences across potexviruses have shown that genetic diversity is not strictly related to or shaped by host type [5,22,29]. This was evidenced by host range experiments where neither SenMV nor AltMV were able to infect papaya, despite being genetically close to BabMV and PapMV, which cause symptomatic infections in papaya. On the other hand, BabMV was unable to infect *Senna alata* and caused symptomless infections in *S*. *occidentalis*. An AlkB domain present in a small group of potexviruses, including BabMV, did not reveal a host related evolutionary pattern either. A co-relation between AlkB containing viruses and their perennial hosts was observed and—given the assumed RNA methylation repair function of this domain—it was hypothesized that continuous exposure to methylation damage in perennials would have been a driver, at some point, for the acquisition of an AlkB domain by the virus genome [17]. It remains unknown, however, the conditions under which this domain would still operate in non-perennial infecting potexviruses such as SenMV and AltMV among others. The lack of clustering among all AlkB containing potexviruses—also observed in other genera within the *Alpha-* and *Betaflexiviridae—*even suggests separate AlkB acquisition events in plant viruses [16].

Despite the genetic and even biological differences between BabMV, PapMV and AltMV, the three viruses were serologically indistinguishable as was evidenced by the detection of BabMV using Agdia-developed antibodies for PapMV and AltMV.

In babaco, virus-like diseases have not been studied in the last three decades. In 1989, a potexvirus was reported from symptomatic babaco in Italy. Based on immune-electron microscopy techniques, it was speculated that the virus was a strain of PapMV [4]. Considering that babaco was introduced to Italy from Ecuador, it is not unreasonable to think that BabMV may correspond to an isolate of the virus originally reported in Italy [4].

Nevertheless, the babaco production in Ecuador has increased significantly in the last two decades; as expected, virus-like diseases have also become conspicuous in the major production areas. Therefore, a certification program for production of virus-free babaco plants needs to be implemented considering *i)* the vegetative propagation nature of this hybrid and *ii)* the use of BabMV-susceptible rootstock, such as chamburo (*V*. *pubescens*), for management of soil-borne pathogens through grafting.

The characterization of BabMV is the first attempt to highlight the importance of virus identification and development of effective detection assays for the implementation of virus-clean certification schemes for this crop.

## Supporting information

S1 Fig Phylogenetic tree. Maximum likelihood inference based on the coat protein. Virus names abbreviations and NCBI acc. numbers are shown.
(TIF)

S2 Fig Electron micrographs. Transmission electron microscopy photographs of virus particles observed in plants infected with babaco mosaic virus.
(PDF)

S1 Table Virus list. List of potexvirus species used for phylogenetic relationships based on the polymerase. NCBI acc. numbers and corresponding references are shown.
(DOCX)
