# Supplementary figures and images for: Characterization of a not so new potexvirus from babaco (Vasconcellea x heilbornii)
Source: PLoS One. 2017 Dec 15;12(12):e0189519. doi: 10.1371/journal.pone.0189519 (PMC5731686; doi:10.1371/journal.pone.0189519)

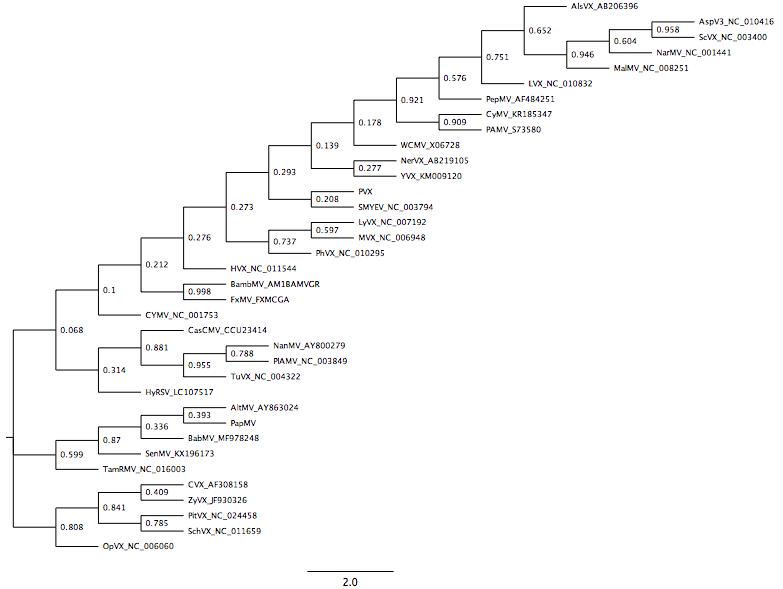

Supplement: S1 Fig — Maximum likelihood inference based on the coat protein. Virus names abbreviations and NCBI acc. numbers are shown. (TIF) [file pone.0189519.s001.tif]
