# Supplementary material for: Characterization of a not so new potexvirus from babaco (Vasconcellea x heilbornii)
Source: PLoS One. 2017 Dec 15;12(12):e0189519. doi: 10.1371/journal.pone.0189519 (PMC5731686; doi:10.1371/journal.pone.0189519)
Supplement: S2 Fig — Transmission electron microscopy photographs of virus particles observed in plants infected with babaco mosaic virus. (PDF) [file pone.0189519.s002.pdf]

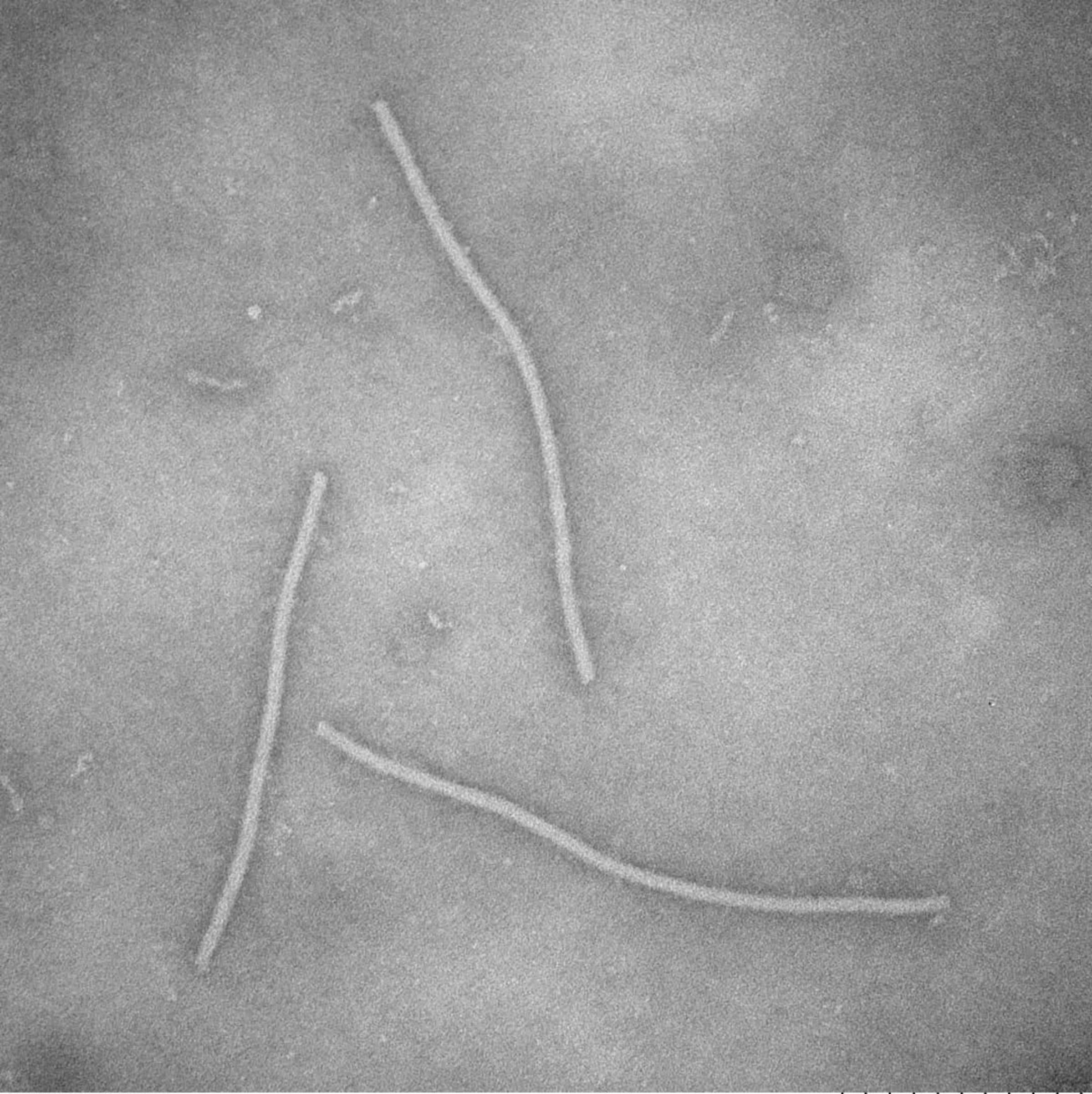

200nm

File name=58 Potex.tif
Image comment=GD, 3 min sample, 1%PTA at pH 6.7
Image date=2017/09/22 14:39:18
Image number=0058
Calibration=3.100
Magnification=x70.0k
Lens mode=Zoom-1

Spot number=5
Image rotation=0°
Acc. voltage=80.0kV
Emission=10.0µA
Stage X=708 Y=-112 Tilt=-0.2 Azim=0.0

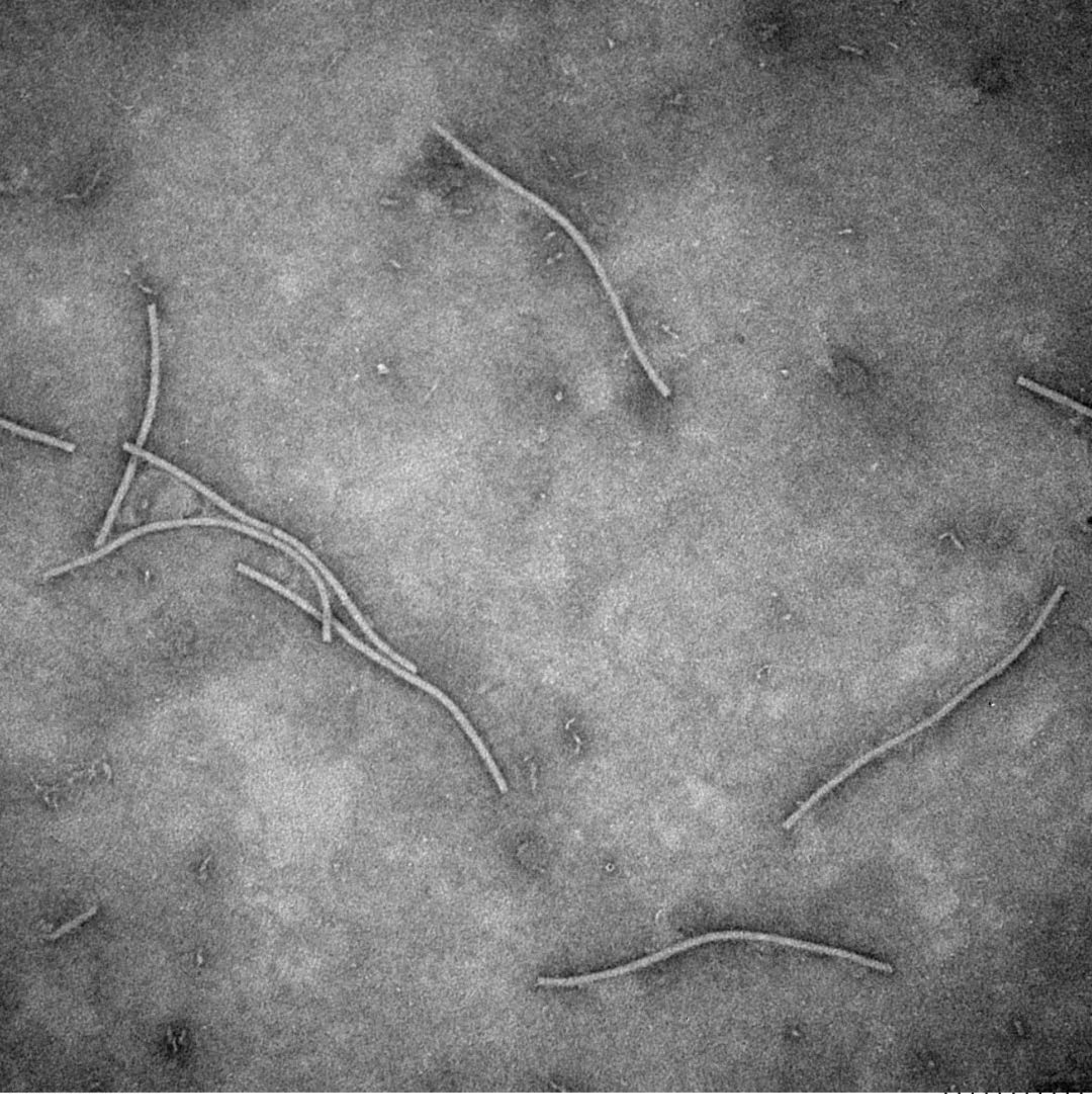

File name=47 Potex.tif
Image comment=GD, 3 min sample, 1%PTA at pH 6.7
Image date=2017/09/22 14:19:05
Image number=0047
Calibration=3.100
Magnification=x40.0k
Lens mode=Zoom-1
Spot number=5
Image rotation=0°
Acc. voltage=80.0kV
Emission=10.0µA
Stage X=683 Y=-341 Tilt=-0.2 Azim=0.0
