# Supplementary material for: Characterization of a not so new potexvirus from babaco (Vasconcellea x heilbornii)
Source: PLoS One. 2017 Dec 15;12(12):e0189519. doi: 10.1371/journal.pone.0189519 (PMC5731686; doi:10.1371/journal.pone.0189519)
Supplement: S1 Table — List of potexvirus species used for phylogenetic relationships based on the polymerase. NCBI acc. numbers and corresponding references are shown. (DOCX) [file pone.0189519.s003.docx]

**Supplemental table 1.** List of viruses used for phylogenetic inferences.

| **Virus name** | **Abbreviation** | **NCBI Acc. number** | **Reference** |
| --- | --- | --- | --- |
| Alstromeria virus X | AlsVX | AB206396 | Fuji et al. 2005 |
| Alternathera mosaic virus | AltMV | AY863024 | Hammond et al. 2006 |
| Asparagus virus 3 | AspV3 | NC_010416 | Hashimoto et al. 2008 |
| Babaco mosaic virus | BabMV | MF978248 | This study |
| Bamboo mosaic virus | BamMV | AM1BAMVGR | Lin et al. 1994 |
| Cactus virus X | CVX | AF308158 | Liou et al. 2004 |
| Cassava common mosaic virus | CsCMV | NC_001658 | Calvert et al. 1996 |
| Clover yellow mosaic virus | CYMV | NC_001753 | Si et al. 1990 |
| Cymbidium mosaic virus | CymMV | KR185347 | Cho et al. 2016 |
| Foxtail mosaic virus | FMV | AY121833 | Bancroft 1991 |
| Hosta virus X | HVX | NC_011544 | Choi et al. 2012 |
| Hydrangea ringspot virus | HdRSV | LC107517 | Yusa et al. 2016 |
| Lettuce virus X | LeVX | NC_010832 | Dizadji et al. 2008 |
| Lily virus X | LVX | NC_007192 | Chen et al. 2005 |
| Malva mosaic virus | MalMV | NC_008251 | Cote et al. 2008 |
| Mint virus X | MVX | NC_006948 | Tzanetakis et al. 2006 |
| Nandina mosaic virus | NaMV | AY800279 | Hughes et al. 2005 |
| Narcissus mosaic virus | NMV | NC_001441 | Zuidema et al. 1989 |
| Nerine virus X | NVX | AB219105 | Fuji et al. 2006 |
| Opuntia virus X | OpVX | NC_006060 | Koenig et al. 2004 |
| Papaya mosaic virus | PapMV | NC_001748 | Sit et al. 1989 |
| Pepino mosaic virus | PepMV | AF484251 | Aguilar et al. 2002 |
| Phaius virus X | PhVX | NC_010295 | Kawakami et al. 2008 |
| Pitaya virus X | PiVX | NC_024458 | Mao et al. unpublished |
| Plantago asiatica mosaic virus | PlAMV | NC_003849 | Solovyev et al. 1994 |
| Potato aucuba mosaic virus | PAMV | S73580 | Xu et al. 1994 |
| Potato virus X | PVX | M72416 | Skryabin et al. 1988 |
| Scallion virus X | ScVX | NC_003400 | Che et al. 2002 |
| Schlumbergera virus X | SchVX | NC_011659 | Koenig et al. 2004 |
| Senna mosaic virus | SenMV | KX196173 | Rezende et al. 2017 |
| Strawberry mild yellow edge virus | SMYEV | NC_003794 | Jelkmann et al. 2002 |
| Tamus red mosaic virus | TRMV | NC_016003 | Menzel and Winter 2012 |
| Tulip virus X | TVX | NC_004322 | Yamaji et al. 2001 |
| White clover mosaic virus | WCMV | X06728 | Forster et al. 1988 |
| Yam virus X | YVX | KM009120 | Mambole et al. 2014 |
| Zygocactus virus X | ZVX | JF930326 | Mao et al. unpublished |
